# Prevalence and Associated Factors of Adolescent (15–19 Years) Childbearing in Ghana

**DOI:** 10.1155/2024/3237882

**Published:** 2024-08-07

**Authors:** Abdul Rauf Alhassan, Tina Wepeamo Wepeba, Kasim Abdulai, Rhubamatu Iddrisu, Gifty Apiung Aninanya

**Affiliations:** ^1^ Department of Surgery Tamale Teaching Hospital, P.O. Box TL 16, Tamale, Ghana; ^2^ Hasbi Research Consultancy, Tamale, Ghana; ^3^ Ghana Organization for Maternal and Child Health (GOMaCH), Tamale, Ghana; ^4^ Nursing and Midwifery Training College, P.O. Box Gu 13, Gushegu, Northern Region, Ghana; ^5^ Department of Clinical Nutrition and Dietetics School of Allied Health Sciences University of Cape Coast, Cape Coast, Ghana; ^6^ Nurses and Midwives Training College, Tamale, Ghana; ^7^ Department of Health Services Policy Planning Management and Economics School of Public Health University for Development Studies, Tamale, Ghana

**Keywords:** adolescent, childbearing, Ghana, predictors, pregnancy

## Abstract

**Background:** Adolescent pregnancies continue to be a global issue that affects more high-income, middle-income, and then low-income countries, with the latter experiencing the majority of cases.

**Aim:** The current study looked into the prevalence and variables predicting adolescent childbearing in Ghana.

**Methodology:** Data from the Ghana Multiple Indicator Cluster Survey (MICS) 2017–2018 was used to conduct an analytical cross-sectional study. The results were examined with SPSS Version 20 (IBM Corp., 2011, and NY). Pearson's chi-square and binary logistics analyses were done for associations. A *p* value of 0.05 was used to determine the analysis's statistical significance.

**Results:** The total number of adolescents isolated from the 2017 Ghana MICS dataset for this study analysis was 2974. The mean age of the study participants was 16.9 ± 1.4 years with a modal age of 15 years. The prevalence of adolescent childbearing according to this study analysis was 12.3%. The predictive factors for adolescent childbearing were increasing age, decreasing educational level, Volta regional originality, ethnic originality of the study participants, and low economic status.

**Conclusion:** The prevalence of adolescent childbearing in this study was significant and needs the attention of all. Programs to improve adolescent reproductive health must take into account multiple levels of elements, such as the individual, family, community, institutions, national, and international challenges that have an impact on such programs.

## 1. Introduction

Anyone aged between 10 and 19 is considered an adolescent according to the World Health Organization (WHO) [1]. The WHO considers individuals between the ages of 10 and 24 when defining young people. The risks and realities of teenage motherhood are well known, with effects beginning with pregnancy and childbirth and continuing throughout the mother's and child's lives. Adolescent pregnancies continue to be a global issue that affects more high-income, middle-income, and then low-income countries, with the latter experiencing the majority of cases [2].

Teenagers in poor nations confront several difficulties such as restrictive laws and policies that limit the use of contraceptives based on a woman's age or marital status, service providers' prejudiced treatment of adolescents' sexual health needs, a lack of autonomy to ensure proper and consistent contraceptive use, lack of information, access to transportation, and financial resources [3]. Many different factors can affect adolescent childbearing, and they can differ from one country or location to another. However, these elements can also be influenced by the parents of the affected girls, their friendships with their peers, and their parents' socioeconomic and educational position [4].

Globally, 16 million girls between the ages of 15 and 19 and 1 million girls under the age of 15 give birth each year, with a teen birth rate of 49 live births per 1000 women [2, 5]. The biggest cause of death for young women [6–10] is problems during pregnancy, childbirth, and postpartum (42 days following childbirth) [11].

Low-income countries have the greatest birth rates (97 live births per 1000) while high-income countries have the lowest rates (12 live births per 1000 teenage girls) [12]. Regionally, the rate of adolescent births is highest in Africa (99 live births per 1000), while it is lowest in the WHO Western Pacific Region (14 live births per 1000) [1]. The results of studies on “parent/child sexual communication and adolescent pregnancy risk” have been uneven, but they have consistently shown that females with a family history of teenage childbearing are at considerably higher risk of teenage pregnancy and childbearing themselves [13].

Ghana's population is younger than most other countries, with about 57% of people under the age of 25. During the 1980s and 1990s, its overall fertility rate drastically decreased but has recently reached a plateau of about four children per woman [14]. In Ghana, teenage pregnancy rates are high (17.8%), and 1 in 5 and 1 in 20 girls get married before turning 18 and 15, respectively [15]. According to studies done in Ghana, teens gave birth to nearly 30% of all babies recorded [16]. In 2014, teenage pregnancy climbed from 13% to 14%, with 16.7% of occurrences happening in rural Ghana and 11.5% in urban Ghana [5].

Although the rate of adolescent pregnancy has decreased globally, it is still rising in emerging nations, with sub-Saharan Africa showing the greatest prevalence [2]. The sexual and reproductive health of young adolescent males and girls between the ages of 10 and 14 is also little understood, as is their fertility. The topic is challenging to investigate methodically due to the cultural sensitivity of the subject, the fact that young girls under the age of 15 are often less sexually active than older adolescents or women, and the fact that they very seldom have children at such young ages [17]. Therefore, the current study looked into the prevalence and variables predicting adolescent childbearing in Ghana among the age group 15–19 years.

## 2. Materials and Methods

### 2.1. Study Design and Data Source

Data from the Ghana Multiple Indicator Cluster Survey (MICS) 2017–2018 was used to conduct an analytical cross-sectional study. Through a formal request, this study's researcher was able to get the 2017–2018 Ghana MICS data from UNICEF. UNICEF gave its consent for the anonymized data to be used. In all ten of Ghana's previous administrative regions, the 2017/2018 MICS used computer-assisted personal interviewing (CAPI) to gather data on population and health indicators. The Ghana Health Service Ethics Review Committee gave its approval to the MICS protocols for the years 2017–2018. All adults who participated in the study verbally agreed to participate before any questionnaires were given out. Consent was sought from parents or guardians for participants between the ages of 15 and 17. All participants received guarantees regarding their free will to leave the interview at any time, as well as voluntary participation, information confidentiality, and anonymity.

### 2.2. Population and Sampling

The 2010 Ghanaian Population and Housing Census (PHC) was used as the sampling frame. Ghanaians between the ages of 15 and 49 were the target demographic for the 2017/2018 MICS. To choose the participants, a two-stage sampling procedure was used. First, 660 enumeration areas/clusters were chosen proportionally to size from the 2010 PHC list. The selection of 13,202 households for the next step of the hiring process required rigorous random sampling, out of which 12,886 were chosen for interviews. Women in the chosen households between the ages of 15 and 49 were eligible to participate in the women's survey. In the chosen households, exactly 14,609 women were found, and 14,374 of them were questioned, for a response rate of 98.4%. Fieldworkers who had received training collected the data. Data collection lasted from 15 October 2017 to 15 January 2018, inclusive.

For the purpose of this current study, all females between the ages of 15 and 19 years, who remained in certain homes the night before the survey (2974), whether they were tourists or permanent residents, were used for this study analysis.

### 2.3. Study Variables

#### 2.3.1. Dependent Variable

The main dependent or outcome variable in this study is adolescent childbearing, thus adolescents with childbirth history.

#### 2.3.2. Independent Variables

The independent variables included the participants' socioeconomic characteristics such as age, education status, region, residence type, marital status, health insurance status, functional difficulties, and wealth index quintile status. Also included as independent variables are participant exposure to the Internet and media such as newspaper, radio, television (TV), computer or tablet, and Internet use.

### 2.4. Statistical Analysis

The results were examined with SPSS Version 20 (IBM Corp., 2011, and NY). The effects of categorical variables were explained using frequency and percentage tables. The chi-square test was used to ascertain how the dependent and independent variables related to one another. A binary logistic regression model was used to determine the predictive variables of adolescent childbearing in Ghana. A *p* value of 0.05 was used to determine the analysis's statistical significance.

### 2.5. Ethical Consideration

The Ghana MICS 2017/2018 dataset was approved for use in this study by the MICS team from UNICEF. Since this analysis needed a secondary look at a dataset without revealing the identity of the participants and their houses, ethical approval was not necessary.

## 3. Results

### 3.1. Characteristics of Study Participants

The total number of adolescents isolated from the 2017 Ghana MICS dataset for this study analysis was 2974. The mean age of the study participants was 16.9 ± 1.4 years with a modal age of 15 years. Most (96.3%) of the study participant had some form of educational attainment with more than half (57.1%, *n* = 2865) of them with a JHS/JSS level of education. The region with the dominant (13.6%) representation was the Ashanti Region, and mostly (37.5%) of the respondents were Akans. More than half (53.9%) of the study participants were from urban communities. The majority (92.3%) of the participants were not in any form of union. About half (54.1%) of the study participants had health insurance. Only 5.1% (*n* = 1128) of the participants had functional difficulties. Economically participants' distribution was almost even across the index wealth quintile categories, though higher among the poorest (26.9%) and middle (20.0%) (Table 1).

### 3.2. Respondents' Exposure to the Internet and Media

There was zero frequency of newspaper or magazine reading among the majority (84.3%) of the participants. Most (42.5%) of the participants were not listening to the radio. Only 32.4% of the participants were not at all exposed to watching TV. The majority (76.1%) of the participants never used a computer or tablet before. And Internet ever use was only 14.8% (*n* = 2849) among the study participants (Table 2).

### 3.3. Socioeconomic Predictors of Adolescent Childbearing

The prevalence of adolescent childbearing according to this study analysis was 12.3%. The regions with the highest prevalence of adolescent childbearing were 17.6% and 17.2% for the Eastern Region and Volta Region, respectively. And the lowest prevalence was recorded among adolescents in the Northern Region (Table 3). The first chi-square analysis was done to identify factors associated with the dependent variable (adolescent childbearing). At this stage, all the studied socioeconomic characteristics except functional difficulties had a significant association with adolescent childbearing with chi-square analysis (Table 4). Also, exposure to the Internet and media factors such as frequency of newspaper or magazine reading and computer and Internet use experience was significantly associated with adolescent childbearing (Table 5).

The predictors of adolescent childbearing were identified using a binary logistics regression model. This was done using the variables' significant association at the chi-square analysis stage. The age of the adolescent predicted childbearing; as their ages increased from 15 to 19 years, the likelihood of childbearing increased (*p* < 0.05). Increasing educational status was a protective factor against adolescent childbearing. Those with JHS/JSS educational level were 59% less likely to engage in adolescent childbearing as compared to those with primary educational level (AOR = 0.41, 95% C.I. = 0.29–0.59). Again with education, those with senior secondary education were 90% less likely to engage in adolescent childbearing when compared to those with primary educational levels (AOR = 0.10, 95%, C.I. = 0.05–0.17). In terms of regional prediction, those from the Volta Region were 131% more likely to engage in adolescent childbearing when compared to those from the Western Region (AOR = 2.31, 95% C.I. 1.04–5.12). With marriage, those not in unions were 93% less likely to engage in adolescent childbearing when compared to those in married unions (AOR = 0.07, 95% C.I. = 0.04–0.14). The ethnic originality of the study participants had a significant association with adolescent childbearing. Those from the Ewe ethnic group were 54% less likely to engage in adolescent childbearing as compared to those from the Akan ethnic group (AOR = 0.46, 95% C.I. = 0.25–0.86). Also, those of the Guan ethnic group were 88% less likely to engage in adolescent childbearing as compared to those from Akan ethnic group (AOR = 0.22, 95% C.I. 0.08–0.60). Again, adolescents from the Mole Dagbani tribe were 53% less likely to bear children as compared to those from the Akan tribe (AOR = 0.47, 95% C.I. = 0.15–0.94). More so, those with Grusi ethnic background were 96% less likely to engage in adolescent childbearing as compared to those from the Akan tribe (AOR = 0.04, 95% C.I. = 0.15–0.94). The other tribes unlisted in this study were 76% less likely to engage in adolescent childbearing as compared to the Akan ethnic group (AOR = 0.26, 95% C.I. = 0.13–0.52). Finally, in this study, better economic status of adolescent girls influences their childbearing; those from the fourth wealth index quintile were 48% less likely to engage in adolescent childbearing as compared to those from the poorest quintile (AOR = 0.52, 95% C.I. = 0.30–0.89). Also, those from the richest quintile were 66% less likely to engage in adolescent childbearing as compared to the poorest quintile (AOR = 0.34, 95% C.I. = 0.18–0.66) (Table 6).

## 4. Discussion

Regionally, the rate of adolescent births is highest in Africa (99 live births per 1000), while it is lowest in the WHO Western Pacific Region (14 live births per 1000) [1]. Adolescent pregnancy rates are very high. Thirty percent (30%) of all births recorded in Ghana in 2014 were by adolescents, and 14% of teenagers between the ages of 15 and 19 had started having children [18]. In this current study, the prevalence of adolescent childbearing according to this study analysis was 12.3%. Trends in the proportion of teenagers (15–19 years) who had begun childbearing decreased from 22% in 1993 to 13% in 2008 and then increased to 14% in 2014 [19]. In this study, prevalence of adolescent childbearing is even lower as compared to the global prevalence of 14% from the UNICEF report [6]. However, this is still very significant given their vulnerability to health consequences of pregnancy and child delivery [6].

Many different factors can affect adolescent childbearing, and they can differ from one country or location to another [4]. In this present study, all the studied socioeconomic characteristics except functional difficulties had a significant association with adolescent childbearing with chi-square analysis. Also, exposure to the Internet and media factors such as frequency of newspaper or magazine reading and computer and Internet use experience was significantly associated with adolescent childbearing. However, the independent predictive factors identified were increasing age, decreasing educational level, regional originality, ethnic originality of the study participants, and low economic status.

The age of the adolescent predicted childbearing; as their ages increased from 15 to 19 years, the likelihood of childbearing increased. Maybe this is so because the increasing age of the adolescent is associated with sexual activeness and fertility [17].

Also, increasing educational status was a protective factor against adolescent childbearing in this study. Those with JHS/JSS educational levels were 59% less likely to engage in adolescent childbearing as compared to those with primary educational levels. Again, with education, those with senior secondary education were 90% less likely to engage in adolescent childbearing when compared to those with primary educational levels. This is in line with an earlier study in Sunyani Municipality of Ghana where being in school predicted the risk of adolescent pregnancy as compared to those not in school [7]. Also, according to Nguyen, Chengshi, and Farber, girls in households with higher formal education have a lower adolescent pregnancy rate [8]. Again, according to research done in West and Central Africa, a girl's educational level and geographic location have a strong positive correlation with adolescent pregnancy [9]. This may due to the fact that higher educational attainment is associated with higher knowledge on reproductive services.

Location is a significant demographic factor that makes difference in our health issues. Adolescent pregnancy can be influenced positively or negatively by the community in which one lives. A constructive conversation about sex is prohibited and seen as evil because so many traditional houses in Ghana are highly conservative [10]. With this present study, in terms of regional prediction, those from the Volta Region were 131% more likely to engage in adolescent childbearing when compared to those from the Western Region. Meanwhile, this did not transfer to the ethnicity of the participants. The dominant tribe in Volta Region is Ewe, but Ewes were 54% less likely to engage in adolescent childbearing as compared to those from the Akan ethnic group. The possible explanation is that the Volta Region is a multiethnic and multilingual, including groups such as the Ewe, the Guan, and the Akan people.

More so, the ethnicity of the adolescent can influence them getting pregnant. In the United State, both non-Hispanic Black teens' (25.8%) and Hispanic teens' (25.3%) birth rates in 2019 were more than twice as high as non-Hispanic White teens' rate (11.4%). The greatest birth rate (29.2%) among all racial/ethnic groups was among American Indian/Alaska Native teenagers [20]. Also, in this study, ethnic originality of the study participants had a significant association with adolescent childbearing. Those from the Ewe ethnic group were 54% less likely to engage in adolescent childbearing as compared to those from the Akan ethnic group. Also, those of the Guan ethnic group were 88% less likely to engage in adolescent childbearing as compared to those from Akan ethnic group. Again, adolescents from the Mole Dagbani tribe were 53% less likely to bear children as compared to those from the Akan tribe. More so, those with Grusi ethnic backgrounds were 96% less likely to engage in adolescent childbearing as compared to those from the Akan tribe. The other tribes not listed in this study were 76% less likely to engage in adolescent childbearing as compared to those Akan ethnic groups. However, this finding negates the finding of an earlier study that was done in Sunyani Municipality Ghana; their study indicated no significant association between ethnicity and adolescent pregnancy [7]. This may be due to the fact that their study was not national study and has to do municipality.

In addition to the factors identified in this study, the better economic status of adolescent girls influences their childbearing; those from the fourth wealth index quintile were 48% less likely to engage in adolescent childbearing as compared to those from the poorest quintile. Also, those from the richest quintile were 66% less likely to engage in adolescent childbearing as compared to that from the poorest quintile. This supports a previous study in Sunyani Municipality of Ghana that found that adolescents with lower socioeconomic status had a 4.1 times higher chance of adolescent pregnancy than those with better socioeconomic status [7]. Young girls from low-income families are more likely to have children as teenagers, according to research by Moore, Jones, and Meador. The authors concluded that an adolescent girl's living circumstances are a strong indicator of her likelihood of getting pregnant at an earlier stage of development [21]. Also, in A.R. Alhassan, Abdulai, and M.A. Alhassan's study in Ghana, lower wealth status was predictor of earlier sexual debut among women [22]. This may be due to the fact that adolescents from low economic background are more likely to exchange sex for favors.

Finally, according to empirical research, teen marriage is a significant predictor of early sexual intercourse and subsequent teen pregnancy [23]. In rural sub-Saharan Africa, where poverty is becoming more common, girls have very few options for their daily survival except from marriage, which can lead to relatively quick marriage transactions with both families. Teenagers in sub-Saharan African traditional cultures have a poor social status, which is portrayed as an economic burden because it prevents them from being employable wage workers [23]. Also, with this current study, those not in unions were 93% less likely to engage in adolescent childbearing when compared to those in a married union. It is obvious in Africa that after marriage, childbearing is what is expected immediately and this explains this finding. And also, those not in marriage relationship were more likely to be involved in abortion practice as it was reported in a Ghana study by Alhassan and Adolipore [24].

Although this study offers essential information about adolescent childbearing, it is not without limitations. Survey results should be regarded cautiously since they cannot fully capture the participants' diverse and nuanced points of view. However, because the data were nationally representative, the study's conclusions can be applied to the whole Ghanaian adolescent population.

## 5. Conclusion

The prevalence of adolescent childbearing in this study was significant that needed the attention of all, given their vulnerability to health consequences of pregnancy and child delivery. Factors such as educational attainments, child marriage, ethnicity, region of originality, and economic status predicted adolescent childbearing. The needs, environment, and history of teenagers should be taken into account while developing interventions and policies. Programs to improve adolescent reproductive health must take into account multiple levels of elements, such as the individual, family, community, institutions, national, and international challenges that have an impact on such programs.

## Figures and Tables

**Table 1:** Socioeconomic characteristics of study participants.

	**Frequency (** **n** = 2974**)**	**Percentage**
Age of woman		
15	698	23.5%
16	533	17.9%
17	615	20.7%
18	583	19.6%
19	545	18.3%
Ever attended school		
Yes	2865	96.3%
No	109	3.7%
Educational achievement (*n* = 2865)		
Primary	515	18.0%
JHS/JSS	1637	57.1%
Secondary/tech/voc/comm	694	24.2%
Higher	19	0.7%
Region		
Western	265	8.9%
Central	297	10.0%
Greater Accra	302	10.2%
Volta	268	9.0%
Eastern	301	10.1%
Ashanti	404	13.6%
Brong Ahafo	264	8.9%
Northern	314	10.6%
Upper East	262	8.8%
Upper West	297	10.0%
Area		
Urban	1372	46.1%
Rural	1602	53.9%
Marital status		
Married	98	3.3%
Cohabitation	131	4.4%
Not in union	2745	92.3%
Ethnicity		
Akan	1114	37.5%
GA/Dangme	225	7.6%
Ewe	347	11.7%
Guan	105	3.5%
Gruma	149	5.0%
Mole Dagbani	642	21.6%
Grusi	143	4.8%
Mande	23	0.8%
Other	226	7.6%
Health insurance		
With insurance	1609	54.1%
Without insurance	1365	45.9%
Functional difficulties (age 18–49 years) (*n* = 1128)		
Has functional difficulty	58	5.1%
Has no functional difficulty	1070	94.9%
Wealth index quintile		
Poorest	800	26.9%
Second	522	17.6%
Middle	595	20.0%
Fourth	530	17.8%
Richest	527	17.7%

*Note:* Data source: GMICS 2017/2018.

**Table 2:** Respondents exposure to Internet and media.

	**Frequency**	**Percentage**
Frequency of reading newspaper or magazine		
Not at all	2508	84.3%
Less than once a week	244	8.2%
At least once a week	190	6.4%
Almost every day	32	1.1%
Frequency of listening to the radio		
Not at all	1264	42.5%
Less than once a week	501	16.8%
At least once a week	636	21.4%
Almost every day	573	19.3%
Frequency of watching TV		
Not at all	963	32.4%
Less than once a week	337	11.3%
At least once a week	628	21.1%
Almost every day	1046	35.2%
Ever used a computer or a tablet		
Yes	711	23.9%
No	2263	76.1%
Ever used Internet (*n* = 2849)		
Yes	422	14.8%
No	2427	85.2%

*Note:* Data source: GMICS 2017/2018.

**Table 3:** Prevalence of adolescent childbearing in Ghana.

	**Ever given birth**		**Total**	**Test statistics**	
	**No**	**Yes**			
Region					
Western	231	34	265	*χ* ^2^	31.175
	87.2%	12.8%	100.0%	df	9
Central	253	44	297	Sig.	< 0.001
	85.2%	14.8%	100.0%		
Greater Accra	276	26	302		
	91.4%	8.6%	100.0%		
Volta	222	46	268		
	82.8%	17.2%	100.0%		
Eastern	248	53	301		
	82.4%	17.6%	100.0%		
Ashanti	352	52	404		
	87.1%	12.9%	100.0%		
Brong Ahafo	228	36	264		
	86.4%	13.6%	100.0%		
Northern	290	24	314		
	92.4%	7.6%	100.0%		
Upper East	240	22	262		
	91.6%	8.4%	100.0%		
Upper West	267	30	297		
	89.9%	10.1%	100.0%		
Total	2607	367	2974		
	87.7%	12.3%	100.0%		

*Note:* Data source: GMICS 2017/2018.

**Table 4:** The association between respondents' socioeconomic characteristics and birth history.

	**Ever given birth**				**Test statistics**	
	**No**		**Yes**			
Age of woman						
15	689	98.7%	9	1.3%	*χ* ^2^	236.270
16	502	94.2%	31	5.8%	df	4
17	550	89.4%	65	10.6%	Sig.	0.000
18	466	79.9%	117	20.1%		
19	400	73.4%	145	26.6%		
Ever attended school						
Yes	2526	88.2%	339	11.8%	*χ* ^2^	18.635
No	81	74.3%	28	25.7%	df	1
					Sig.	0.000
Educational achievement						
Primary	412	80.0%	103	20.0%	*χ* ^2^	77.130
JHS/JSS	1429	87.3%	208	12.7%	df	3
Secondary/tech/voc/comm	666	96.0%	28	4.0%	Sig.	0.000
Higher	19	100.0%	0	0.0%		
Region						
Western	231	87.2%	34	12.8%	*χ* ^2^	31.175
Central	253	85.2%	44	14.8%	df	9
Greater Accra	276	91.4%	26	8.6%	Sig.	0.000
Volta	222	82.8%	46	17.2%		
Eastern	248	82.4%	53	17.6%		
Ashanti	352	87.1%	52	12.9%		
Brong Ahafo	228	86.4%	36	13.6%		
Northern	290	92.4%	24	7.6%		
Upper east	240	91.6%	22	8.4%		
Upper west	267	89.9%	30	10.1%		
Area						
Urban	1254	91.4%	118	8.6%	*χ* ^2^	32.929
Rural	1353	84.5%	249	15.5%	df	1
					Sig.	0.000
Marital status						
Yes, currently married	32	32.7%	66	67.3%	*χ* ^2^	702.557
Yes, living with a partner	42	32.1%	89	67.9%	df	2
No, not in union	2533	92.3%	212	7.7%	Sig.	0.000
Ethnicity						
Akan	958	86.0%	156	14.0%	*χ* ^2^	24.829
GA/Damgme	187	83.1%	38	16.9%	df	8
Ewe	293	84.4%	54	15.6%	Sig.	0.002
Guan	98	93.3%	7	6.7%		
Gruma	136	91.3%	13	8.7%		
Mole Dagbani	579	90.2%	63	9.8%		
Grusi	128	89.5%	15	10.5%		
Mande	23	100.0%	0	0.0%		
Other	205	90.7%	21	9.3%		
Health insurance						
With insurance	1388	86.3%	221	13.7%	*χ* ^2^	6.306
Without insurance	1219	89.3%	146	10.7%	df	1
					Sig.	0.012
Functional difficulties (age 18–49 years)						
Has functional difficulty	43	74.1%	15	25.9%	*χ* ^2^	0.238
Has no functional difficulty	823	76.9%	247	23.1%	df	1
					Sig.	0.626
Wealth index quintile						
Poorest	680	85.0%	120	15.0%	*χ* ^2^	50.060
Second	435	83.3%	87	16.7%	df	4
Middle	512	86.1%	83	13.9%	Sig.	0.000
Fourth	475	89.6%	55	10.4%		
Richest	505	95.8%	22	4.2%		

*Note:* Data source: GMICS 2017/2018.

Abbreviations: *χ*^2^ = chi-square, df = degree of freedom, Sig. = 95% significance level.

**Table 5:** The association between respondents' exposure to Internet and media and birth history.

	**Ever given birth**				**Test statistics**	
	**No**		**Yes**			
Frequency of reading newspaper or magazine						
Not at all	2170	86.5%	338	13.5%	*χ* ^2^	20.492
Less than once a week	229	93.9%	15	6.1%	df	3
At least once a week	176	92.6%	14	7.4%	Sig.	0.000
Almost every day	32	100.0%	0	0.0%		
Frequency of listening to the radio						
Not at all	1095	86.6%	169	13.4%	*χ* ^2^	7.505
Less than once a week	444	88.6%	57	11.4%	df	3
At least once a week	575	90.4%	61	9.6%	Sig.	0.057
Almost every day	493	86.0%	80	14.0%		
Frequency of watching TV						
Not at all	835	86.7%	128	13.3%	*χ* ^2^	7.302
Less than once a week	306	90.8%	31	9.2%	df	3
At least once a week	562	89.5%	66	10.5%	Sig.	0.063
Almost every day	904	86.4%	142	13.6%		
Ever used a computer or a tablet						
Yes	665	93.5%	46	6.5%	*χ* ^2^	29.768
No	1942	85.8%	321	14.2%	df	1
					Sig.	0.000
Ever used Internet						
Yes	394	93.4%	28	6.6%	*χ* ^2^	16.766
No	2091	86.2%	336	13.8%	df	1
					Sig.	0.000

*Note:* Data source: GMICS 2017/2018.

Abbreviations: *χ*^2^ = chi-square, df = degree of freedom, Sig. = 95% significance level.

**Table 6:** Binary logistics regression for predictors of adolescent childbearing.

	**B**	**p** **value**	**AOR**	**95% C.I. for AOR**	
				**Lower**	**Upper**
Age of woman					
15		*p* ≤ 0.001			
16	1.569	*p* ≤ 0.001	4.802	2.211	10.427
17	2.241	*p* ≤ 0.001	9.403	4.525	19.540
18	2.985	*p* ≤ 0.001	19.779	9.591	40.786
19	3.393	*p* ≤ 0.001	29.762	14.336	61.788
Educational achievement					
Primary		*p* ≤ 0.001			
JHS/JSS	−0.889	*p* ≤ 0.001	0.411	0.287	0.588
Secondary/TECH/VOC	−2.350	*p* ≤ 0.001	0.095	0.053	0.171
Higher	−20.453	0.999	0.000	0.000	
Region					
Western		0.010			
Central	0.068	0.828	1.071	0.578	1.984
Greater Accra	0.357	0.358	1.429	0.667	3.063
Volta	0.838	0.039	2.311	1.043	5.120
Eastern	0.352	0.280	1.422	0.751	2.692
Ashanti	0.589	0.054	1.802	0.989	3.281
Brong Ahafo	−0.055	0.871	0.947	0.490	1.831
Northern	−0.813	0.077	0.444	0.180	1.092
Upper East	−0.857	0.078	0.424	0.164	1.100
Upper West	−0.548	0.224	0.578	0.239	1.399
Area					
Urban					
Rural	0.201	0.263	1.222	0.860	1.737
Marital status					
Married		*p* ≤ 0.001			
Cohabitation	−0.395	0.300	0.674	0.319	1.421
Not in union	−2.569	*p* ≤ 0.001	0.077	0.041	0.142
Ethnicity					
Akan		0.002			
GA/Damgme	−0.033	0.910	0.967	0.544	1.720
Ewe	−0.774	0.015	0.461	0.247	0.859
Guan	−1.525	0.003	0.218	0.080	0.595
Gruma	−0.883	0.059	0.414	0.165	1.035
Mole Dagbani	−0.760	0.019	0.467	0.247	0.883
Grusi	−0.979	0.037	0.376	0.150	0.944
Mande	−20.506	0.998	0.000	0.000	
Other	−1.356	*p* ≤ 0.001	0.258	0.127	0.524
Health insurance					
With insurance					
Without insurance	−0.608	*p* ≤ 0.001	0.545	0.404	0.735
Wealth index quintile					
Poorest		0.014			
Second	−0.177	0.422	0.838	0.544	1.290
Middle	−0.262	0.260	0.769	0.488	1.214
Fourth	−0.654	0.017	0.520	0.303	0.891
Richest	−1.071	0.001	0.343	0.178	0.660
Frequency of reading newspaper or magazine					
Not at all		0.795			
Less than once a week	0.180	0.579	1.198	0.633	2.266
At least once a week	0.311	0.362	1.365	0.700	2.660
Almost every day	−17.604	0.998	0.000	0.000	
Ever used a computer or a tablet					
Yes					
No	0.210	0.338	1.234	0.803	1.898
Ever used Internet					
Yes					
No	0.147	0.593	1.158	0.676	1.983

*Note:* Data source: GMICS 2017/2018.

## Data Availability

Data associated with this study is available for users to download/analyze after registering on the United Nations Children's Fund (UNICEF) MICS website as a data user.

## References

[1] WHO (2019). World Health Organization. https://www.who.int/health-topics/adolescent-health#tab=tab_1.

[2] WHO (2020). *Adolescent pregnancy*.

[3] Todd N., Black A. (2020). Contraception for adolescents. *Journal of Clinical Research in Pediatric Endocrinology*.

[4] Neal S., Matthews Z., Frost M., Fogstad H., Camacho A. V., Laski L. (2012). Childbearing in adolescents aged 12–15 years in low resource countries: a neglected issue. New estimates from demographic and household surveys in 42 countries. *Acta Obstetricia et Gynecologica Scandinavica*.

[5] UNESCO (2017). *Early and unintended pregnancy & the education sector; Evidence Review And Recommendations*.

[6] UNICEF (2021). *Early childbearing can have severe consequences for adolescent girls*.

[7] Asare B. Y. A., Baafi D., Dwumfour-Asare B., Adam A. R. (2019). Factors associated with adolescent pregnancy in the Sunyani Municipality of Ghana. *International Journal of Africa Nursing Sciences*.

[8] Nguyen H., Chengshi S., Farber N. (2016). *Prevalence and factors associated with teen pregnancy in Vietnam: results from two national surveys*.

[9] Fenn N. S., Edmeades J., Lantos H., Onovo O. (2015). *Child marriage, adolescent pregnancy and family formation in West and Central Africa: patterns, trends and drivers of change*.

[10] Krugu J. K., Mevissen F., Münkel M., Ruiter R. (2017). Beyond love: a qualitative analysis of factors associated with teenage pregnancy among young women with pregnancy experience in Bolgatanga, Ghana. *Culture, Health & Sexuality*.

[11] Kassa G. M., Arowojolu A. O., Odukogbe A. A., Yalew A. W. (2018). Prevalence and determinants of adolescent pregnancy in Africa: a systematic review and meta-analysis. *Reproductive Health*.

[12] World Health Statistics (2019). *World Health Statistics 2019: monitoring health for the SDGs, sustainable development goals*.

[13] Elizabeth W. W., Leslie L. R., Nathan C. N. (2016). Teenage pregnancy: the impact of maternal adolescent childbearing and older sister’s teenage pregnancy on a younger sister. *BMC Pregnancy and Childbirth*.

[14] Index mundi (2021). Index mundi. https://www.indexmundi.com/ghana/demographics_profile.html.

[15] de Groot R., Kuunyem M. Y., Palermo T. (2018). Child marriage and associated outcomes in northern Ghana: a cross-sectional study. *BMC Public Health*.

[16] Yussif A. S., Lassey A., GYk G., Kantelhardt E. J., Kielstein H. (2017). The long-term effects of adolescent pregnancies in a community in Northern Ghana on subsequent pregnancies and births of the young mothers. *Reproductive Health*.

[17] United Nations, Department of Economic and Social Affairs, Population Division (2020). *Fertility among very young adolescents aged 10-14 years (ST/ESA/SER.A/448)*.

[18] Graphic online (2016). *Teenage pregnancy in Ghana: assessing situation and moving forward*.

[19] GSS, GHS, and ICF International (2015). *Ghana demographic and health survey 2014*.

[20] Martin J., Hamilton B., Osterman M., Driscoll A. (2021). Births: final data for 2019. *National Vital Statistics Reports*.

[21] Moore B., Jones E., Meador J. (2010). Teenage pregnancy rates: a multiple regression analysis of teen pregnancy rates and the correlates for Texas counties in the year 2010. *Politics, Bureaucracy, and Justice*.

[22] Alhassan A. R., Abdulai K., Alhassan M. A. (2021). Early sexual debut among Ghanaian women: correlates and psychological effect. *BioMed Research International*.

[23] UNICEF (2018). *Child marriage: latest trends and future prospects*.

[24] Alhassan A. R., Adolipore J. N. A. (2021). Prevalence and predictive factors of induced abortion among women in Ghana: data analysis of maternal health survey, 2017. *Journal of Clinical Research and Reports*.

